# Antimicrobial Silver in Medicinal and Consumer Applications: A Patent Review of the Past Decade (2007–2017)

**DOI:** 10.3390/antibiotics7040093

**Published:** 2018-10-26

**Authors:** Wilson Sim, Ross T. Barnard, M.A.T. Blaskovich, Zyta M. Ziora

**Affiliations:** 1 School of Chemistry & Molecular Biosciences, The University of Queensland, Brisbane, QLD 4072, Australia; wilson.sim@uq.net.au (W.S.); rossbarnard@uq.edu.au (R.T.B.); 2 ARC Training Centre for Biopharmaceutical Innovation, The University of Queensland, Brisbane, QLD 4072, Australia; 3 Institute of Molecular Bioscience, The University of Queensland, Brisbane, QLD 4072, Australia; m.blaskovich@imb.uq.edu.au

**Keywords:** antibiotic resistance, antimicrobial activity, medicinal silver, patents, silver, silver nanoparticles, synergism

## Abstract

The use of silver to control infections was common in ancient civilizations. In recent years, this material has resurfaced as a therapeutic option due to the increasing prevalence of bacterial resistance to antimicrobials. This renewed interest has prompted researchers to investigate how the antimicrobial properties of silver might be enhanced, thus broadening the possibilities for antimicrobial applications. This review presents a compilation of patented products utilizing any forms of silver for its bactericidal actions in the decade 2007–2017. It analyses the trends in patent applications related to different forms of silver and their use for antimicrobial purposes. Based on the retrospective view of registered patents, statements of prognosis are also presented with a view to heightening awareness of potential industrial and health care applications.

## 1. Introduction

Silver is a soft and shiny transition metal which is known to have the highest reflectivity of all metals [1]. Among its many useful properties, silver it recognized to have antimicrobial activity. Silver is known to be biologically active when it is dispersed into its monoatomic ionic state (Ag^+^), when it is soluble in aqueous environments [2]. This is the same form which appears in ionic silver compounds such as silver nitrate and silver sulfadiazine, which have been frequently used to treat wounds [3]. Another form of silver is its native nanocrystalline form (Ag^0^). The metallic (Ag^0^) and ionic forms can also appear loosely associated with other elements such as oxygen or other metals and can form covalent bonds or coordination complexes [3].

**Figure 1:**
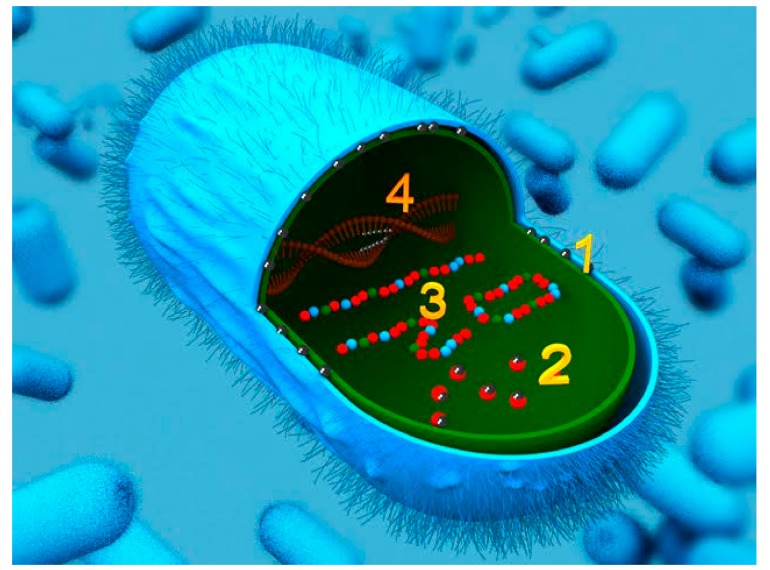
Silver’s action on a bacterial cell. 1. Silver can perforate the peptidoglycan cell wall. 2. Silver inhibits the cell respiration cycle. 3. Metabolic pathways are also inhibited when in contact with silver. 4. Replication cycle of the cell is disrupted by silver particles via interaction with DNA.

To date, there are three known mechanisms by which silver acts on microbes. Firstly, silver cations can form pores and puncture the bacterial cell wall by reacting with the peptidoglycan component [4]. Secondly, silver ions can enter into the bacterial cell, both inhibiting cellular respiration and disrupting metabolic pathways resulting in generation of reactive oxygen species [5]. Lastly, once in the cell silver can also disrupt DNA and its replication cycle [6] (Figure 1). A recently published review includes more details about the bactericidal mechanisms of silver, along with methods of silver nanoparticle preparation [7]. Throughout history, silver has consistently been used to restrict the spread of human disease by incorporation into articles used in daily life. The earliest recorded use of silver for therapeutic purposes dates back to the Han Dynasty in China *circa.* 1500 B.C.E [8]. Silver vessels and plates were frequently used during the Phoenician, Macedonian, and Persian empires [9]. Families of the higher socioeconomic classes during the middle-ages were so acquainted with the usage of silver that they developed bluish skin discolorations known as *argyria*, an affliction which may have led to the term ‘blue blood’ to describe members of the aristocracy [10]. Modern medicine utilizes medical grade forms of silver, such as silver nitrate, silver sulfadiazine, and colloidal silver [11]. 

The discovery of antibiotics in the early 20th century led to a cessation in the development of silver as an antimicrobial agent. However, the development of increasing levels of bacterial resistance to most antibiotics in recent years has led to reexamination of the potential of this ancient remedy [7,12] including studies with patients using colloidal silver and antibiotics [13]. This review aims to demonstrate the wide and ever-expanding applications of silver in medicine, health care, and other daily life activities, with a focus on the patents registered during the past decade. A similar patent review was published in Expert Opinion on Therapeutic Patents in 2005 [14], covering patents compiled from 2001–2004. The current review extends to the years 2007–2017. An analysis of the growth of patents describing antimicrobial silver applications is presented throughout this review, along with commentary of selected examples demonstrating some of the more interesting applications. Our analysis has separated these discussions of the use of silver into four general categories: Medical applications, personal care products, domestic household products, and agricultural/industrial applications.

## 2. Discussion

### 2.1. Antimicrobial Silver for Clinical and Medical Usage

This section presents a selection of some of the most interesting and unique patented products which utilize silver for their bactericidal action in the medical field, including therapies based on silver’s antimicrobial properties. The product numbers referred to in this section correspond to those listed in Table 1.

Surface coatings incorporating silver are a common application. One new approach is a method for producing ready packed medical apparatus which sterilizes itself upon the opening of the package, by creating a vapor that activates a silver-containing hydrophilic surface coating (Product No. 1). The antimicrobial properties of silver have been highly valued in medical application where implanted devices are coated with silver nanoparticles for the antimicrobial effects, but manufacturers need to be aware that this application is claimed in a patent application (Product No. 2) with a very broad claim 1: “An article that is implantable in an animal, the article comprising a microparticulate silver-containing antimicrobial layer stably adhered upon at least one surface of the article.” However, the application does not appear to have progressed towards granting. Invasive surgical tools such as medical grade needles (Product No. 3) can also be coated with silver nanoparticles as described in its related patent (Product No. 4). Medical devices that are directly introduced into the human body that contain silver include vascular catheters (Product No. 5), bone implants (Product No. 6), and biliary duct brackets (Product No. 7). Another topical application of antimicrobial silver has incorporated it into coatings applied to the interior surface of a building's exterior wall (Products No. 8a and 8b).

Another general use is for topical treatments. Numerous topical gels with different formulations of silver have been patented. Silver was first used to treat burn wounds in the form of 0.5% silver nitrate solution and silver sulfadiazine cream in 1960 [35]. However, this was impractical as the dressings required rehydration every couple of hours. To overcome this limitation silver nanoparticle-based gels and silver salt-based gels have been developed (Products No. 9a, 9b and 10), with all approaches still considered novel.

**Figure 2:**
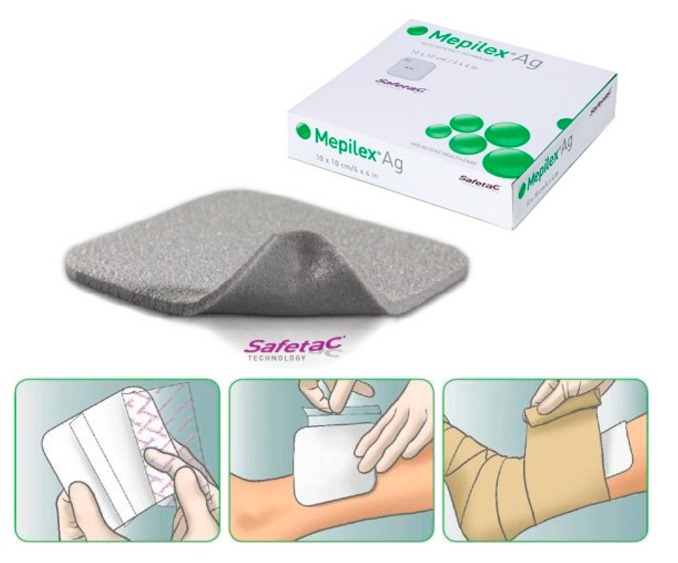
**Mepilex^®^ Ag with instructions for application**. Images used with permission of Mölnlycke Health care, Sweden.

Silver based wound dressings have greatly improved in efficacy compared to standard dressings, and more complex dressings have been developed. New knowledge in burn wound management led to the discovery of a method to immobilize silver nanoparticles on a gel-support matrix which is attached to a wound dressing (Product No. 11). A recently commercialized wound dressing allows a prolonged use of the dressing for up to 7 days or until saturation, without reapplication (Figure 2). It is made possible through its design, which slowly releases silver ions upon contact with wound exudates. Its highly absorbent padding is also coated with a layer of silicone which is aimed to reduce pain during removal and reapplication of the dressing. (Product No. 12). The use of silver with wound dressings is known to reduce scarring and such formulations are widely used (Product No.13). Silver-based wound dressings are available under brand names with different compositions, such as Mepilex^®^ Ag, Acticoat™, Aquacel^®^, Flaminal^®^, Allevyn^®^ Ag, and Biatain^®^ Ag, SILVERCEL™. Other products containing a silver component, not specifically developed for wound healing, have been patented for treatment of bacterial infection (Products No. 14a and 14b). 

Silver has also been applied across the dental field. Silver has been the key component in dental amalgam fillings for more than one hundred years. However, its antimicrobial properties were not patented. Silver is used in the prevention of infection during and after dental surgery (Product No. 15). Dental support fixtures made out of silver and denture materials, and other body restoration objects, having silver nanoparticles as additives can reduce bacterial infections, especially during first few months of installation (Products No. 16 and 17).

### 2.2. Antimicrobial Silver in Personal Care Products

**Figure 3:**
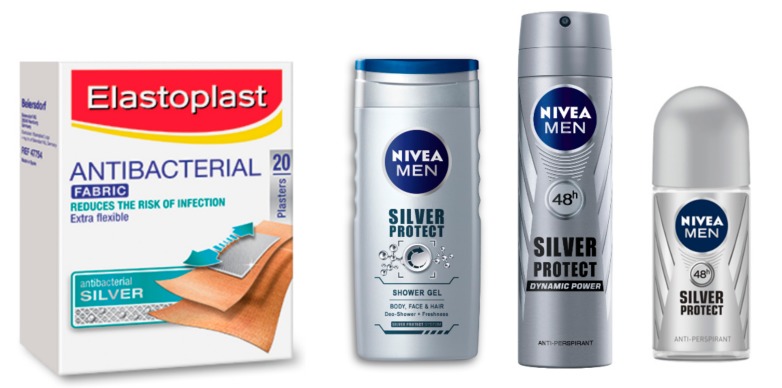
Commercial utilization of silver in consumer product lines, including Elastoplast (First aid bandages) and NIVEA (Shower gels and deodorants) for enhanced antimicrobial properties. *Images used with permission of Beiersdorf AG, Germany.*

This section presents grooming products and devices which utilize silver for its sanitizing effects, summarized in Table 2. Hygiene and grooming products such as shavers (Product No. 18), toothbrushes (Product No. 19) and sanitary pads (Product No. 20) are frequently employed under adverse conditions where they encounter the bacteria microbiome, but are relied upon to be sanitary. One example of such usage is by the German public company “Beiersdorf AG” which has products incorporating silver for its added antimicrobial properties. They have applied silver over a wide range of products from shower gels and deodorants to first aid bandages (Figure 3).

Since using silver to treat skin infections is common, researchers in dermatology frequently resort to silver for treating conditions related to bacterial colonization, such as body odors (Product No. 21), acne outbreaks (Product No. 22), eczema and rash (Product No. 23). 

A range of other personal health products have also added silver to improve their hygienic capacities, including contact lenses (Product No. 24), antimicrobial fabric garments (Products No. 25a, 25b and 25c), breast pump assemblies (Product No. 26), and hair dye (Product No. 27).

Most cosmetic products come in the form of cream, aqueous lotions, or hydrogel medium. It is observed that most manufacturers favor the incorporation of silver colloids into their products as they do not precipitate and separate, with the added benefit of acting as a preservative. Colloidal silver is defined as a mixture of silver ions and silver nanoparticles suspended in an aqueous medium. They are usually synthesized by electrolysis using a set of silver cathodes [48]. Colloidal silver was first used in 1891 by a surgeon named B.C Crede to sterilize wounds [9]. The use of silver grew in popularity between 1900 to the 1940s. Subsequently, antibiotics supplanted the use of silver [9]. Today, many products are offered not only as colloidal silver solutions, but also as personal devices suitable for home use, that synthesize colloidal silver. However, the commercialization of colloidal silver has been accompanied by inconsistencies in colloidal silver production and properties, as well as cases of unexpected side effects. Therefore, the Food and Drug Administration (FDA) has excluded any commercialized colloidal silver that claims health benefits without scientific evidence [49]. Similar action has been taken by the Therapeutic Goods Administration (TGA) in Australia [49] and the European Commission (EC) [50]. The commercial sales of colloidal silver are not banned, but claims of health benefits without scientific support are not permitted.

### 2.3. Antimicrobial Silver in Domestic Products

The antimicrobial applications of silver started in ancient times in domestic products like silver plates and pitchers [9]. With that in mind, there continue to be domestic applications of silver, particular for surface treatments (Table 3). 

Silver is widely incorporated into surface coatings of electrical goods such as automated bathtubs (Product No. 28), laundry washing machines (Product No. 29), air purifiers with silver filters (Product No. 30) and refrigerators (Product No. 31), to produce ‘bacteria-free’ products. Application of silver nanoparticles to other household objects with frequent handling such as keyboards (Product No. 32), bath safety aids (Product No. 33), and bathroom safety handles (Product No. 34). Special stand-alone products such as containers for meat or water/wine/milk storage (Products No. 35a and 35b) are useful applications where bacterial contamination may present a health issue. 

Despite the many beneficial innovations in the use of silver as an antimicrobial agent, its application in cleaning products and disposable tools such as gloves (Product No. 36), disinfectant wipes (Product No. 37), and cleaning detergent (Product No. 38) may have negative environmental impacts. Cleaning products, once used, usually end up in sewage treatment systems, and eventually the environment. This is a concern for silver nanoparticles, as there are currently no effective methods for filtering out silver nanoparticles. The release of large amount of silver products into the environment may lead to disturbances of the microbiological ecosystem, and potentially lead to bacterial resistance to silver [63]. Consequently, alternative methods of sanitization should be considered such as the application of alcohol or bleach which are sufficient for domestic purposes, or employing ‘fixed’ silver containing surfaces that reduce the risk of environmental release.

Apart from being a threat to beneficial environmental bacteria, another issue to be addressed is the possible longer-term reduction of the potency of silver in killing microbes. Since the discovery of antibiotics, the efficacy of antibiotics has been compromised by over-prescription and over-usage, leading to the current antibiotic crisis. The presence of low levels of antibiotics in the environment fosters the generation of multiple drug resistant strains [64]. Silver is not immune to the generation of bacterial resistance, with several reports in recent years [65,66]. This history suggests a need for a systemic reassessment of the usage of silver in domestic products, so that it is not used too extravagantly, or released haphazardly. 

### 2.4. Antimicrobial Silver in Agricultural and Industrial Products 

Silver has also been used for a variety of agricultural and industrial products. In industry, large scale water purification can be made cost effective by using colloidal silver for purification as it is needed only in small quantities and can purify large quantities of water, though potential environmental risk needs to be considered [67]. For agriculture use, silver has been incorporated in nylon ropes that are used to tie down plants, cover them with netting, and for various other applications. These ropes normally decay after time due to bacterial biofilm formation, so the silver prevents this decomposition [68]. Agricultural use of silver products must be carefully assessed to avoid any impact on the microbial flora and symbiosis. The growth of healthy crop plants relies heavily upon the formation of symbiotic microbes around the roots such as nitrifying bacteria and mycorrhiza [69]. Studies have shown that the contact of bioactive silver to nitrifying bacteria impedes the formation of symbiotic channels [70].

Table 4 presents patented industry and agricultural related products utilizing silver as an antimicrobial agent. An example of agricultural use is Product No. 39 which uses Ag (I) and Ag (II) to treat infections in plants, while Products No. 40 and 41 with coating of a single rope strand with silver to prolong resistance to biofilm formation. Silver coatings can be beneficial in industrial machines which require a completely sterile environment to manufacture food or medical grade products, employing silver in the parts that come in direct contact with the products (Product No. 42). Machinery parts are usually designed for prolonged periods and incorporating silver particles into these materials provides an effective means of isolation and retention of silver so that it is not released into the environment easily.

Polymers are extremely versatile and when impregnated with silver nanoparticles, they can be used for numerous applications, such as mass-produced food storage containers (Product No. 43) and industrial scale waste bins (Product No. 44). Sterility in the food and therapeutics industry is crucial, so the incorporation of silver into manufacturing equipment in contact with consumer products can be regarded as an appropriate usage. However, in the case of daily used food containers, frequent usage of silver may not be ideal as there is a risk of accumulation in the human body if the silver leaches, potentially leading to similar side effects as were observed in the middle ages when silver utensils were frequently used [9].

Products No. 45a and 45b describes a water filtration unit containing immobilized silver nanoparticles for water purification purposes. The invention and manufacture of industrial cleaning solutions containing silver (Product No. 46) is a potentially widespread application, as there is a need for instant effective sanitization to prevent bacterial transmission. However, precautions must be observed to prevent environmental release.

### 2.5. Overview of Patent Literature from 2007–2017

In the previous sections, applications of antibacterial silver in a variety of fields were discussed. This section presents an overview of silver-related patents on a global level for the purpose of understanding the trends, major applications as well as major contributors. Methods by which the data sets were obtained are reported in Section 3.

**Figure 4:**
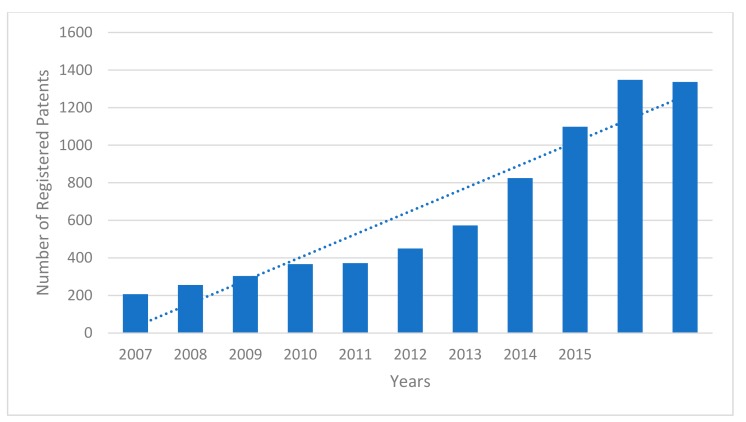
Trend analysis of yearly number of patent registrations involving antimicrobial silver applications over the past decade.

Tracking the number of patents disclosing antimicrobial applications of silver for each year over the past decade, as summarized in Figure 4, shows that there has been a steady upward trajectory in the number of silver patent applications in recent years. The increase may have reached a plateau in 2016/17, but it will be necessary to consider data from 2018 and onwards to confirm this hypothesis. 

**Figure 5:**
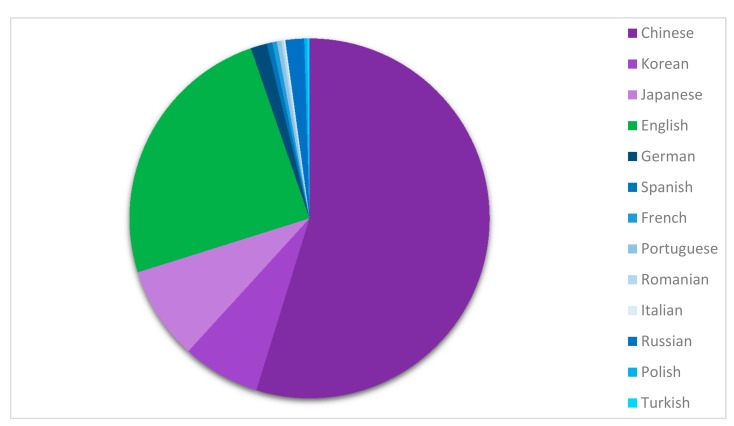
Analysis of language of patent registrations involving antimicrobial silver over the past decade with major contribution of Chinese (55%), English (25%), Korean (7%), Japanese (8%), and others (5%).

The data obtained from Figure 4 can be further dissected to reveal patents registered under each language, as shown in Figure 5. This can be linked to a deduction of the country of origin of these patents. This analysis demonstrates that patents claiming antimicrobial silver products are predominantly contributed by Asian countries, with China (55%), Korea (7%), and Japan (8%) comprising 70% of the chart. Patents registered in English compose 25% while another 5% are various European language patent registrations. It has been speculated that since the FDA and EMA have reduced influence in the Asian countries [49], this opens up opportunities in Asia for innovations with antimicrobial silver. Indeed, there is some basis for this speculation given the large number of silver related patents being registered in Asian languages, but additional research will be needed to establish the causes of this phenomenon. One of the potential reasons explaining the more substantial contribution of Asian countries in patented silver related innovations and consumer products is the fast-track approval pathway for new drugs in China, supported by consumer trials that are significantly cheaper than in other countries, which can be performed on higher number of participants. There is also a Chinese government strategy to commercialize non-Chinese ideas in China and financially support innovators who are willing to patent their ideas in China, with a preference to procure products whose IP is owned or registered in China. Finally, the database search may produce multiple results for a single patent that are particularly difficult to detect for Chinese applications because Chinese individuals’ names typically have only three or two characters [79]. 

**Figure 6:**
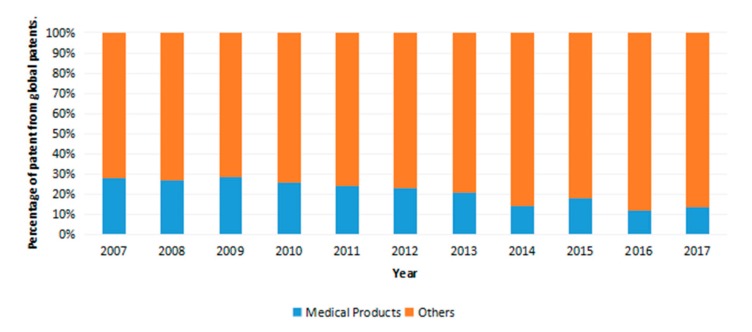
Total patents registered globally for silver used in the medical field compared to all other applications.

We have also assessed the application of silver according to its field of use. Based on the analysis from Figure 6, only about 20% of the patents in each year claimed medical uses of silver. This could mean that approximately about 80% of the patented silver applications are for other usages, such as domestic, agricultural and industrial usage. If this trend continues, there is potential for damage to the ecosystem if the non-medical uses result in release to the environment. This could lead to a worrisome situation where bacteria evolve resistance to silver, one of few promising alternatives to current classes of antibiotics.

## 3. Materials and Methods 

The patent analysis data and trend chart were generated through search results obtained from the scientific publication database, SciFinder^®^ by the American Chemical Society. It was accessed through the University of Queensland Library portal. Results generated were accurate as of 15 June 2018.

### 3.1. Dataset 1—Application of Antibacterial Silver from the Global Perspective

The keywords used to generate this search were “antibacterial + silver” and “silver + medical” that narrowed down the number of silver related patents to those presenting the word “silver” in the title and describing only silver components. Therefore, patents with the general terms such as “metal nanoparticles” covering all nanomaterials with potential antibacterial applications like silver, platinum, gold, palladium, copper, zinc, and other metals, are not included here. Result limiters used were publication years (2007–2017) and document type (Patent). Search results yielded 5054 hits of exact words and concepts related to its words. No duplicates were found throughout the result set. The full result was analyzed by publication year and was sorted out by natural order to reflect results in yearly order. Obtained results were used to generate a bar chart as Figure 5 by using Microsoft Word chart sketching function. 

### 3.2. Dataset 2—Application of Antibacterial Silver from a Regional Perspective

The keywords used to generate this search were as for Dataset 1, “antibacterial + silver”. Result limiters used were publication years (2007–2017) and document type (Patent). Search results yielded 5054 hits of exact words and concepts related to its words. No duplicates were found throughout the result set. The full result was analyzed by language and was sorted out by frequency of region. Results obtained were used to generate the pie chart (Figure 6) by using the Microsoft Word chart sketching function.

### 3.3. Dataset 3—Application of Antibacterial Silver in the Medical Field

The keywords used to generate this search were “silver + medical”. Result limiters used were publication years (2007–2017) and document type (Patent). Search results yielded 1310 hits of exact words and concept related to its words. No duplicates were found throughout the result set. The full result was analyzed by publication year and was sorted out by natural order to reflect results in yearly order. Results obtained were tabulated against dataset 1 to in order to obtain a 100% stacked bar chart presented at Figure 6 by using Microsoft Word chart sketching function.

## 4. Conclusions

The use of silver for its antimicrobial properties is increasing in numerous fields, including the medical, consumer, agricultural and industrial sectors. In just over 10 years, nearly 5000 new applications have been registered. The majority of the patents are from Asian countries, with Chinese language applications representing more than 50% of the global total, followed by Korean and Japanese language filings. Only about 20% of patents are registered in English.

While the potential benefits of silver are attracting increased attention, a number of publications have pointed out potential adverse effects from the overuse of silver, such as ecosystem disturbance [80], and bacterial resistance to silver [81]. Since our “armory” of antibiotics has been depleted by the rise in antimicrobial resistance, silver represents a new hope, but mindful use must be considered at an early stage to prevent a repetition of past mistakes.

We suggest that the application and commercialization of silver related products should be critically reassessed to avoid, or at least minimize, these adverse effects. In particular, while incorporation of silver products in an enclosed environment is justifiable, products which are expected to release silver into the environment should be avoided. There is ample evidence [82] that there can be adverse long-term effects from consumption or exposure to silver, so silver products should only be used in circumstances where (1) there is an absolute need for it, such as a medical intervention, and (2) in modes where silver is immobilized and containable.

## Figures and Tables

**Table 1:** List of patented medical grade products and medically related products containing silver as an antimicrobial agent.

No.	Patent Title	Brief Product Description	Patent Number	Filing Date	Ref.
1	Ready to use medical device with instant antimicrobial effect	A medical apparatus packaging designed to activate a bioactive silver coating and other bactericidal elements upon opening of package.	US20150314103A1	5 November 2015	[15]
2	Use of silver-containing layers at implant surfaces	The method of coating medical implants with various forms of silver for infection prevention.	US20130344123A1	4 March 2013	[16]
3	Medical needles having antibacterial and painless function	Surgical needles coated with silver nanoparticles for the prevention of infection.	WO2006088288A1	5 January 2006	[17]
4	Fracture-setting nano-silver antibacterial coating	A method to coat invasive medical instruments with silver nanoparticles.	CN203128679U	3 April 2013	[18]
5	Antimicrobial closure element and closure element applier	The coating of the internal structure of a vascular portal device and the interior of its applicator.	US20080312686A1	9 June 2008	[19]
6	Bone implant and systems that controllably release silver	A specially designed bone implant which allows surgeons to control the release of silver ions.	WO2012064402A1	19 May 2012	[20]
7	Nanometer silver antibacterial biliary duct bracket and preparation method thereof	A biliary duct implant bracket made from plastic coated uniformly with silver nanoparticles to prevent biofilm formation at site of implant.	CN102485184A	3 December 2010	[21]
8a	Antimicrobial coatings on building surfaces	A coating with antimicrobial silver applied to the interior surface of a building’s exterior wall.	US7641912B1	5 January 2010	[22]
8b			US8282951B2	9 October 2012	[23]
9a	Silver nanoparticle dispersion formulation	A topical gel to treat dermal infections with 1% w/w silver nanoparticles as active ingredient.	WO2007017901A2	16 February 2007	[24]
9a			EP3359166A1	15 August 2018	[25]
10	Antimicrobial silver hydrogel composition for the treatment of burns and wounds	An aqueous gel with a range of silver salt as its active ingredient made for the treatment of wounds specifically caused by burns.	WO20120282348A1	5 May 2011	[26]
11	Polysaccharide fibers for wound dressings	The method of coating wound dressing with a gel matrix where silver can be immobilized and applied to a wound to aid healing.	WO2013050794A1	5 December 2012	[27]
12	Antimicrobial, silver-containing wound dressing for continuous release	Wound dressing capable of releasing silver ions to aid healing upon contact with fluids from the wound.	US20070286895A1	24 August 2007	[28]
13	Nano-silver wound dressing	A wound dressing with enhanced antimicrobial properties for improved scarring.	US20070293799A1	9 December 2008	[29]
14a	Metal containing materials	Silver containing materials for treatment of bacterial conditions.	US8425880B1	23 April 2013	[30]
14b			US7255881B2	14 August 2007	[31]
15	Dental Uses of Silver Hydrosol	Silver suspended in aqueous gel used to reduce infection risks of dental procedures.	US9192626B2	24 November 2015	[32]
16	Antimicrobial silver nanoparticle additive for polymerizable dental materials	Denture material made with the addition of silver nanoparticles for additional antimicrobial effect.	US20070213460A1	13 September 2007	[33]
17	Silver ion coated products for dental and other body restoration objects	Silver coating with antimicrobial, antifouling and deodorant properties	US20180245278A1	30 August 2018	[34]

**Table 2:** List of patented personal-care products containing silver as an antimicrobial agent.

No.	Patent Title	Brief Product Description	Patent Number	Filing Date	Ref.
18	Cosmetic and /or medical device for antimicrobial treatment of human skin with silver particles	Technology in which shaving devices can deposit silver ions unto skin in place of traditional antiseptic medium.	DE102012224176A1	26 June 2014	[36]
19	Antimicrobial thermoplastic polyurethane for toothbrush and preparation method for antimicrobial thermoplastic polyurethane	Addition of silver nanoparticles into plastic materials which are used to manufacture bristles of tooth brushes.	CN103254401A	28 April 2013	[37]
20	Sanitary towel capable of removing peculiar smell and manufacturing method thereof	Infusion of silver nanoparticles into fibers of sanitary pads which prevents the growth of odor causing microbes on menstrual discharges.	CN102961778A	21 November 2012	[38]
21	Solid oil cosmetics containing antimicrobial Ag zeolites and aluminum chlorohydrate	Deodorants and topical creams for the prevention of odor causing bacteria.	JP2013071914A	22 April 2013	[39]
22	Antimicrobial agents containing fine silver particle-carrying polypeptides and daikon radish fermentation products, and cosmetics containing them	The invention of a cosmetic lotion preservative consisting of silver nanoparticles.	JP2010059132A	18 March 2010	[40]
23	Functional cosmetic including nano silver	Addition of silver nanoparticles into manufactured cosmetics for its antimicrobial effect which aids in the prevention of acne and pimples.	KR20070119971A	21 December 2007	[41]
24	Antimicrobial contact lenses and methods for their production	Contact lenses manufactured from materials infused with silver nanoparticles for antimicrobial effects.	US20030044447A1	6 March 2003	[42]
25a	Silver-containing antimicrobial fabric	Textile material manufactured from fibers embedded with silver nanoparticles.	US20050037057A1	17 February 2005	[43]
25b			US7754625B2	13 July 2010	[44]
25c			WO2018160708	7 September 2018	[45]
26	Breast pump assemblies having an antimicrobial agent	Suction cup segment of device coated with silver ion exchange resin to prevent possible microbial contamination into breast milk.	US20080139998A1	12 June 2008	[46]
27	Nano-silver inorganic antibacterial nutritional hair dye	Hair colorant having additional silver nanoparticles as preservatives.	CN104224617A	27 August 2014	[47]

**Table 3:** List of patented home-use products containing silver as an antimicrobial agent.

No.	Patent Title	Brief Product Description	Patent Number	Filing Date	Ref.
28	Automatic cleaning system for bathtub or piping	A cleaning system attached to a Silver ion generator which flows through hot water inlet pipe which sanitizes bathtubs as a self-cleaning function.	JP2009268576A	19 November 2009	[51]
29	Clothes washing machine	Laundry washing machine consisting of a silver ion generator which will be released during each wash cycle.	US20080041117A1	21 February 2008	[52]
30	Air purifier, useful for neutralizing bad smells	An electronic air cleaning device which draws unclean air through an immobilized silver filter killing any airborne odor causing bacteria.	DE102007040742A1	3 March 2009	[53]
31	Antibiotic method for parts of refrigerator using antibiotic substance	Distribute silver ions within the fridge to slow the spoilage of food spoilage.	US7781497B2	24 March 2010	[54]
32	Submersible keyboard	A waterproof and washable keyboard with key caps made from plastic embedded with silver ions.	US20090262492A1	22 October 2009	[55]
33	Bactix silver-based antimicrobial additive in bath aids	Bath safety aids made from silver impregnated polymers for long lasting antimicrobial effects.	US20130029029A1	31 January 2013	[56]
34	Bacteria-resistant grab bar	Disability support bar paddings made out of silicone rubber impregnated with silver nanoparticles as antimicrobial additives.	US20100148395A1	17 December 2008	[57]
35a	Antimicrobial reusable plastic or glass container	Collapsible food storage containers mainly for meat or water/wine/milk storage in kitchen composing of an antimicrobial silver fabric as its bottom inner layer.	US20070189932A1	10 February 2006	[58]
35b			WO2018137725A1	2 August 2018	[59]
36	Nano-silver antibacterial gloves	Domestic latex gloves impregnated with silver nanoparticles and other antimicrobial elements.	CN202738872U	20 February 2013	[60]
37	Natural silver disinfectant compositions	General surface cleaner containing soluble silver salt for added antimicrobial effect.	US20100143494A1	10 June 2010	[61]
38	Antiseptic solutions containing silver chelated with polypectate and EDTA	Laundry liquid having aqueous suspension of colloidal silver as additive for its antimicrobial properties.	US7311927B2	25 December 2007	[62]

**Table 4:** List of patented industry and agricultural related products utilizing silver as an antimicrobial agent.

No.	Patent Title	Brief Product Description	Patent Number	Filing Date	Ref.
39	Method and compositions for treating plant infections	Method of applying high valency silver to treat infection in plants of the *Rosaceae* family.	US20120219638A1	21 November 2011	[71]
40	Silver yarn, plied yarn silver yarn, functional fabric using same, and method for producing	Strong weather resistant rope for agricultural purposes made with polyester and silver-plated fiber yarn to prevent growth of biofilms.	CN102439205A	2 May 2012	[72]
41	Silver coated nylon fibers and associated methods of manufacture and use	The method and manufacture of industrialized antimicrobial fabric woven from nylon fibers impregnated with silver.	US20100166832A1	1 July 2010	[68]
42	Rolling apparatus having plastic parts containing antibacterial and antifungal silver (oxides)	Industry scale food grade rollers made from silver impregnated plastic for better hygiene and disease prevention.	JP2005201385A	28 July 2005	[73]
43	An antimicrobial food package	Food grade polymer containers with interiors coated with silver nanoparticles to prolong food freshness.	WO2014001541A1	28 June 2013	[74]
44	Rotationally molded plastic refuse container with microbial inhibiting inner surface and method	Industry scale plastic garbage container interiorly lined with silver nanoparticles for improved waste treatment.	US20080185311A1	7 August 2008	[75]
45a	Sustained silver release composition for water purification	Water filtration unit containing immobilized silver nanoparticles for water purification purposes.	WO2012140520A8	23 March 2012	[76]
45b			US20180186667A1	5 July 2018	[77]
46	Method for Producing Antimicrobial Agent Micro-Particle	An industry cleaning liquid having silver nanoparticles as its active ingredients.	JP2007161649A	28 June 2007	[78]
